# Superabubic Prostatectomy

**Published:** 1917-08

**Authors:** Nathaniel P. Rathbun

**Affiliations:** New York


### SUPRAPUBIC PROSTATECTOMY

**By Nathaniel P. Rathbun, M.D., F.A.C.S.,**
**New York.**

(From the Urological Department of the Brooklyn Hospital.)

Practically every operation in surgery has gone through a process of evolution, various operators contributing suggestions from time to time until a more or less standardized course of procedure has been perfected. More often than not progress has been made along the line of simpler methods. These observations apply particularly to the operation of prostatectomy. In the early days the plan of attack was crude and complicated, the mortality and morbidity were extremely high, and the results obtained in the few survivors were often poor. Today it is not unusual to find a series of a hundred cases reported with one or two deaths or no deaths at all, and the results obtained in a properly managed case are among the most gratifying in the whole field of surgery. It is fair to say that one or two per cent. is not a normal expression of the present mortality. In fact, I believe that even now the mortality varies from four to twenty per cent., depending largely upon the skill of the individual operator.

Of course, it is almost unnecessary to say that much of the progress made is due to a more intelligent selection of cases; much credit, however, is due to improvements in preparation, operation, and aftercare. While the principles involved in these procedures are more or less along the same general plan, there are numerous minor variation, and I doubt if any two operators proceed along identical lines. This paper is a brief synopsis of the methods employed by the writer in the average case and is based upon a small personal series of fifty-four operations. Nothing original is claimed other than my two combination of steps in what appears to me the most desirable manner.

With a few notable exceptions, such as Young, of Baltimore, I believe most operators select the suprapubic route, employing the perineal mode of attack when specially indicated. Given a patient in whom prostatectomy seems to be indicated— this paper does not consider the indications and contraindication—the first problem which presents itself is that of preliminary preparation. I believe that in every instance the bladder should be drained and that in the majority of cases this can be most satisfactorily accomplished through an indwelling catheter. I can see no advantage in the so-called two step method except when special indications exist. I reserve this procedure for three types of cases: those with vesical calculi, patients in whom it is impossible to pass a soft carether into the bladder, and in those cases unable to tolerate a catheter after it is inserted. All of these, and particularly the two last groups are relatively infrequent.

If the bladder is much distended, as it often is, the catheter is passed into the bladder and four ounces of urine withdrawn. A clamp is then placed upon the catheter and it is anchored in the bladder. Every two hours the clamp is removed, another four ounces of urine withdrawn until the bladder is emptied. After this the clamp is permanently detached and the urine is allowed to flow continuously into some convenient receptacle. All this time the patient is allowed to be up and about and is given a light diet and an abundance of water. The bowels are kept open. He is given one dose of 0.5 c. c. of mixed stock vaccines, and urotropin and sodium benzoate, of each ten grains three times a day. The kidney function is tested every two or three days. I rely for this upon the blood urea test, the first, second, and third, hour phthalein excretion, and the twenty-four hour urea. The bladder is irrigated daily with a two per cent. boric acid solution. The blood pressure is recorded, the heart and lungs examined, and where indicated, appropriate treatment is instituted for any trouble that may be noted. The length of time for draining the bladder varies from three to fourteen days or more, depending upon various conditions, the kidney function, condition of the bladder, etc. A badly infected bladder with atonic walls may require a rather long preliminary drainage, whereas a relatively clean bladder will require but a few days.

The objects of preliminary drainage are threefold: 1, gradually to remove back pressure from the kidney, thus avoiding the dangerous renal congestion that might follow the sudden release of obstruction; 2, to help clean up a badly infected bladder and permit the muscle to regain its tone, and 3, in clean bladders as Cabot and Crabtree have recently pointed out, to vaccinate the patient against a gross infection that might, and probably always does occur at the time of the prostatectomy.

The patient having been prepared and contraindictions eliminated, we are ready to proceed with the operation. On the morning of the day preceding the removal of the gland, the patient receives a dose of castor oil and another injection of stock vaccines. On the morning of the operation he receives no food, but is encouraged to drink water up to within one hour of the time he is sent to the operating room. One half hour before going to the operating room he is given a hypodermic injection of morphine, the dose varying from one-eighth to one-fourth grain depending upon the size and vigor of the individual. No anesthetic is administered before he is brought to the operating room and placed upon the table in position for operation. The bladder is again irrigated and finally distended with a two per cent. solution of boric acid and the catheter is removed. The field of operation is prepared as usual with sterile sheets, towels, etc. The operator is prepared in the usual manner, except that the right hand is not gloved, and an extra large sterile sheeve is drawn over the gloved hand and gown. In the meantime no ether has been given. We then proceed to infiltrate the line of incision with cocaine using a solution of one to 1,000. After the first puncture of the needle, ether is started by the drop method on an open mask. The cocaine solution is injected first into the skin and then under the skin along the line of incisions. The skin is then divided and the aponeurosis and muscle infiltrated and opened. About thirty c. c. of cocaine solution is usually employed. The prevesical fat is carefully incised and the peritoneal fold recognized and pushed back as far as possible toward the summit of the bladder. At this point there is occasionally a slight resistance on the part of the patient. The bladder is then punctured as near the summit as possible, the fluid evacuated, and the opening in the bladder stretched sufficiently to admit two fingers. Two fingers of the gloved hand are then passed into the rectum and the prostate and bladder neck pushed well up into the pelvis. The index finger of the right hand is gradually forced into the internal sphincter as far as the first joint, thus dilating the muscle and pushing it out of harm's way. The mucous membrane is then nicked with the finger at the upper left quadrant, the line of cleavage carefully sought for and the gland enucleated and delivered usually in two pieces. At this point the ether is discontinued. The amount required is usually two or three ounces. The sleeve and glove are then removed from the left hand and the operation completed with ungloved hands.

Following the enucleation, no irrigation, hot or otherwise is employed. With one exception, of which I shall speak later, hemorrhage, primary or secondary, has never given me the slightest concern. I believe if the proper line of cleavage is followed and the prostate cleanly enucleated and the bladder cavity left strictly alone, Nature will take care of the bleeding by her usual methods of muscle contraction, vessel retraction, clot, etc.

The bladder wound is snugly closed around a rubber drainage tube at the upper angle of the incision, using interrupted chromic sutures. The aponeurosis is closed with a continuous chromic suture leaving a cigarette drain in the space of Retzius, bringing the rubber tube out through the same opening at the lower end of the wound. The skin is approximated with sutures of interrupted silkworm gut. By this time the patient is usually conversing and the whole procedure has required ten or fifteen minutes. There has been no shock, very little bleeding, and but small amount of anesthesia.

The patient is now returned to his bed and tap water given by the Murphy drip method until he is able to take water freely by the mouth. This is usually the afternoon of the same day. He is encouraged to move about in bed from the first and sit up in bed on the day following. He is usually out of bed on the seventh day. Occasionally, for various reasons, we find it wise to get these patients out a little earlier than this. The cigarette drain is removed in forty-eight hours and replaced by a small wick of iodoform gauze. On the day after the operation the urotropin and sodium benzoate are again started and the diet gradually increased according to indications. No irrigations are employed for the first two weeks and often not at all. I believe in the first few days irrigations may dislodge clots and start secondary bleeding, and when employed a few days later emboli are apt to be thrown off into the general circulation. The rubber drainage tube is removed on the seventh day and the patient instructed to try to pass urine every hour or two during the day. Primary union of the rest of the wound is the rule. Many methods have been suggested for collecting the urine during the interval between the time the drainage tube is removed and the time when the patient is passing his urine in the natural way. I have found nothing more satisfactory than the simple expedient of laying a piece of rubber dam two feet square upon the abdomen, cutting out a small circle about three inches in diameter over the sinus, spreading zinc oxide ointment over the skin and cut edge. Several absorbent pads are applied over the wound and the rubber dam is folded over the pads. Of course these pads must be changed several times a day, and with competent nursing the patient's bed clothes are never wet. On about the fourteenth day it is my custom to pass a No. 30 French sound into the bladder. I believe this may prevent contractures which occasionally occur at the bladder neck following prostatectomy.

The average time of complete closure of the suprapubic sinus is twenty-four days. The earliest complete closure in my series is seventeen days; the longest, forty-three days. When the wound is very slow in closing, a soft catheter may be passed through the urethra and tied in place for a few days and the wound strapped and otherwise encouraged in healing. In this event, it is well to use a bladder irrigation once daily. Of course there are many minor variations from the procedure as outlined, but it seems to me to fit very well for the routine cases.

In my series there were four deaths which can be directly attributed to the operation and two of them preventable. These last two may be charged to lack of experience in early cases.

One patient died from a severe infection of the prevesical space, complicated by uncontrolled hiccoughs, due, I have no doubt, to pericystitis and localized peritonitis. This might have been prevented by better operative technic and more intelligent aftercare. One man died from hemorrhage. This was a case of carcinoma of the prostrate and bladder neck in which the prostate was dug out piecemeal. In spite of all measures taken to control it, oozing continued for several days, with final exhaustion and death. The patient should either have not been operated on at all or should have had a radical excision of the prostate and bladder neck in one piece.

Another patient died from moderate renal insufficiency complicated by bedsores, exhausion, etc. This patient was a man eighty-six years of age with marked arteriosclerosis, rather poor kidney function, and complete obstruction. He was a poor subject for operation and a correspondingly poor prognosis was given. The operation, however, was undertaken at the earnest solicitation of the patient, his physician and his relatives.

The other death was somewhat of a surprise and was hard to explain. A man sixty-one years of age had a moderately large prostate complicated by a large vesical calcus. The operation was performed in two stages, the calculus being removed at the first stage and the prostate one week later. His cardiovascular system was apparently in good shape and his phthalein excretion normal. Yet, following the enucleation there was complete urinary suppression and he died in forty-eight hours.

There were two other deaths which I do not believe could be fairly attributed to the operation. One was a man sixty-six years old with marked arterioscerosis and aortic regurgitation. On the fifth day, while apparently making a perfectly smooth convalescence, he sat up in bed while drinking a cup of coffee and suddely fell forward and was dead. The other, a man seventy-five years old, with a double mitral lesion, died suddenly while sitting up in a chair on the fourteenth day. I do not think that the operation hastened the death of these patients, but they must be included in mortality statistics.

Of the patients who survived, all had a smooth convalescence, with the exception of three with epididymitis, and the end results were uniformly good. Including the sudden deaths noted, my mortality from the fifty-four cases was eleven per cent. While this is not very commendable, I am confident that with increased experience, a more intelligent selection of cases, and continued employment of the methods outlined, I shall be able to make a much better showing with my next half hundred cases.—New York Medical Journal.

### ADVANTAGES OF GERMICIDAL SOAP.

On solution in water Germicidal Soap (McClintock) liberates a small quantity of free alkali. This prevents the coagulation of albumin and permits the mercuric iodide contained in the soap to thoroughly penetrate bacterial and tissue cells.

Germicidal soap is a valuable disinfectant in surgery, in gynecology, in obstetrics, and in routine practice. It is not only detergent, but it is a penetrating antiseptic at the same time. It is an excellent lubricant for sounds and catheters. It is always ready for use. No weighing or measuring is necessary. There is no waste. Hands, instruments and field of operation are quickly disinfected with the one material.

Germicidal Soap does not attack nickled or steel instruments, as does bichloride of mercury. It will not cause numbing of the hands as does carbolic acid.

Germicidal Soap is supplied in two strengths: Germicidal Soap, two per cent. mercuric iodide—large cakes, one in a carton; Germicidal Soap, Mild, one per cent. mercuric iodide—large cakes, one in a carton—small cakes, five in a carton; Germicidal Soap, Soft, one per cent., in collapsible tubes; and Germicidal Soap Surgical, one per cent., in cylindrical cakes wrapped in perforated paper and enclosed in a nickel-plated case. It is well to specify "P. D. & Co." in ordering.

